# Chronic Catarrhal Appendicitis in School Children

**Published:** 1915-02-27

**Authors:** 


### THE SCHOOL DOCTOR AND SLIGHT AILMENTS.

### Chronic Catarrhal Appendicitis in School Children.

At routine inspections the school doctor not infrequently meets with children who present a series of signs and symptoms which, although seemingly vague and unimportant in themselves, are, as experience often shows, of decided importance and gravity. The children are generally of the type which the mothers and teachers style "delicate"; in other words, they present the definite "habitus asthenicus" of Stiller. They have smooth, semi-transparent skins, pasty complexions, are much pigmented, easily fatigued, show slight signs of nervous inco-ordination, and have probably swollen cervical glands, oral sepsis, and septic or enlarged tonsils. Moreover, they present a history of recurring digestive or intestinal trouble, diarrhœa alternating with constipation, slight attacks of vomiting, comparatively frequent bilious attacks, and vague abdominal pains. On closer examination, in the majority of cases, certain more definite signs are elicited. The child, for instance, has definite, though slight, tenderness on deep pressure in the right iliac region; on carefully pressing upwards along the course of the descending colon slight pain may be elicited in the right iliac region—a modification of Rovsing's sign—or an extreme flexion and extension of the right thigh, similar slight pain is complained of in the region of the right hip. In some cases there is a slight difference in the size of the child's pupils, the one on the right side being larger than the one on the left.

### The Question of Operation.

Children who show these signs should be carefully watched, as potential or actual sufferers from chronic catarrhal appendicitis. At the first examination inquiry should be made as to whether or not the child has had anything in the nature of an attack of abdominal pain accompanied with griping pain or with flatulence or vomiting. Where there is a definite history of such attacks a diagnosis of chronic catarrhal appendicitis and a recommendation to have an operation done are fully justified by experience. Chronic catarrhal appendicitis in children is an insidious disease, which is apt to develop with startling rapidity, and the best and safest course is to advise surgical treatment as soon as possible, even when there is some doubt as to the certainty of the diagnosis. An operation for appendicectomy in a child, performed during the latent stage of the disease, is almost devoid of risk to life, and its after-results, under proper treatment, are admirable.

The differential diagnosis is, however, often a matter of some difficulty, even when the train of symptoms is well marked. Chronic constipation in children is perhaps the most common condition that simulates appendicitis; probably the former is only a phase of the latter, since in both the main symptoms in the early stages are those due to intestinal toxæmia of a slight degree. The acute attacks may be due to ordinary gripes, caused by eating indigestible food, to worms, or, much more rarely, to constitutional disease, such as purpura, or to the onset of some infectious disease, such as measles or scarlet fever. Worms are so common in children, and their effects upon asthenic children are often so marked, that in the first place the treatment should be directed to removing any possible source of error that may be due to their presence. The old-fashioned quassia enema is the best and surest safeguard that can be used for this. If worms are present it generally reveals their presence in an unmistakable manner. If the enema, properly administered, has no effect, it may be taken that the child does not suffer from worms. Worm tablets and cakes are of comparatively little use, and where the presence of a tapeworm is suspected more drastic medication is called for. It is worth while to bear in mind that several German authorities have recently laid stress on helminthiasis as a probable cause of chronic catarrhal appendicitis, and that the two conditions may, and often do, co-exist.

### A Text-Book Oversight.

Too much stress can scarcely be laid on the fact that the ordinary text-book descriptions of appendicitis in children are descriptions not of chronic catarrhal appendicitis, but of commencing peritonitis. In children, if one has to await the onset of vomiting, rise of temperature, quickened pulse-rate, rigidity of the right rectus muscle, actual diarrhœa, or acute abdominal pain, one runs a grave risk of having to undertake the responsibility of treating a case of localised peritonitis with commencing abscess formation. These chronic cases flare up with amazing suddenness in children. In one case a boy of eleven was noted down as a probable case of chronic catarrhal appendicitis for following up. He had several of the vague signs given above, and had suffered from recurrent attacks of tonsillitis, and from at least one attack of abdominal pain accompanied with gastric disturbance. The mother was advised to seek surgical treatment, and, in view of the fact that her husband had died of acute appendicitis, was readily induced to take the child to a London hospital for advice. There she was reassured and told that the child was delicate; a tonic was prescribed and operation was declined. Not satisfied with this opinion, she tried a second institution, where the surgeon suggested that constipation and not appendicitis was the cause of the symptoms, but offered to remove the appendix on the parents' responsibility.

This, however, the mother was not prepared to agree to, and the child was treated for constipation, with immediately good results. A few months subsequently the boy "ricked himself" in carrying a young brother; immediately afterwards he complained of severe abdominal pain, vomited, and became collapsed. An operation was undertaken the same night, when it was found that a fairly large appendicular abscess had ruptured and infected the general peritoneal cavity, with the result that the child died. If school doctors succeed in educating the profession and the public to the grave necessity for surgical interference in these so-called slight and chronic cases, they will have done yeoman service indeed. At present the tendency is to overlook these slight conditions and to await the onset of graver symptoms and signs before calling in the surgeon. It is therefore satisfactory to note that more operations are yearly being done as the result of the school doctor's advice, and that the percentage of fatal cases in children is steadily decreasing.

Something must be said as to the after-treatment in cases which have been operated upon. The immediate results are nearly always excellent. The child rapidly improves in colour and general health. Such improvement must, of course, in part at least, be ascribed to the rest and care in hospital, but much also is due to the removal of the source of infection—namely, the diseased and septic appendix. After a few months, however, the child's general condition is less satisfactory. This is in the great majority of cases due to constipation, which appears invariably to be a sequel, early or late, of appendicectomy. Some attention to the diet—the inclusion of fruit, cooked and uncooked, of whole-meal porridge, and of green vegetables in the dietary—is the most obvious change necessary; and the administration of some mild laxative, such as a few drops of the aromatic syrup of senna, are in general all that is desirable to combat this condition of constipation. Once the bowels have been coaxed into something like regularity, habit and attention to diet will ensure a good result so far as health is concerned. At the same time, it is desirable that any other source of sepsis—for instance, chronically enlarged and septic tonsils—should be removed.

